# Physician Satisfaction With Virtual Ophthalmology Clinics During the COVID-19 Pandemic: A Tertiary Eye Care Center Experience

**DOI:** 10.7759/cureus.23837

**Published:** 2022-04-05

**Authors:** Adi M Al Owaifeer, Samar A Al-Swailem, Abdulaziz M Al Dehailan, Abdulrahman Al Naim, Mohammed F Al Molhim, Rajiv B Khandekar

**Affiliations:** 1 Research Department, King Khaled Eye Specialist Hospital, Riyadh, SAU; 2 Department of Ophthalmology, College of Medicine, King Faisal University, Al Hasa, SAU; 3 College of Medicine, King Faisal University, Al Hasa, SAU; 4 Department of Ophthalmology, King Faisal University, Al Hasa, SAU; 5 Ophthalmology, Faculty of Medicine, University of British Columbia, Vancouver, CAN

**Keywords:** virtual clinic, pandemic, physician satisfaction, teleophthalmology, telemedicine

## Abstract

Background

In this study, we aimed to assess ophthalmologists’ experience with teleophthalmology during the coronavirus disease 2019 (COVID-19) pandemic in the central region of Saudi Arabia. In addition, we evaluated their satisfaction level and explored their satisfaction determinants.

Methodology

We conducted an online survey for ophthalmologists who participated in the virtual ophthalmology clinic during COVID-19 between November 2020 and September 2021. The survey was used to evaluate ophthalmologists’ experience with teleophthalmology during the pandemic. Ophthalmologists were asked to measure their satisfaction with equipment and technical issues, communication, and clinical assessment, and to provide an overall program evaluation. Data were analyzed via frequency measures (e.g., numbers, percentages, mean, and standard deviation).

Results

Out of the 113 ophthalmologists who were invited to participate in our study, 71 completed the survey. In total, 23 (32.4%) participants were general ophthalmologists, 15 (21.1%) were subspecialists in the cornea, 16 (22.5%) were subspecialists in glaucoma, one (1.4%) was a subspecialist in neuro-ophthalmology, seven (9.9%) were subspecialists in pediatric ophthalmology, eight (11.3%) were subspecialists in the retina, and one (1.4%) participant was a subspecialist in oculoplastic. Overall, 56.3% of the respondents were satisfied with teleophthalmology. Ophthalmologists who subspecialized in the retina demonstrated higher levels of satisfaction than other subspecialties. The most common challenge reported by ophthalmologists in the virtual consultation was the lack of adequate equipment to evaluate the patients (53.5%), followed by technical issues (43.7%) and the patients’ lack of experience in using virtual consultation services (38%). Overall satisfaction score was the highest among ophthalmologists who reported providing at least five video consultations before the survey.

Conclusions

The findings from our study suggest that the subspeciality of ophthalmologists and the number of video consultations conducted by ophthalmologists are important determinants in their level of satisfaction with teleophthalmology. The majority of the respondents were satisfied with the virtual clinic during the COVID-19 pandemic. The current pandemic could pave the way for the future use of telemedicine in ophthalmology if virtual eye examinations become standardized.

## Introduction

Ophthalmic care has been altered drastically during the coronavirus disease 2019 (COVID-19) pandemic. Social distancing regulations have negatively affected patient visits and clinic workflow and have limited office-based practices to urgent cases only [1,2]. In-person visits of eye patients declined by 88-97% in comparison to pre-pandemic levels. Patients with diabetic retinopathy, glaucoma, and age-related macular degeneration who require long-term follow-up could not be managed effectively, resulting in a risk of irreversible vision loss [1,3]. To lower the risk of sight-threatening conditions, medical practices adopted remote delivery of healthcare services via telemedicine [4-7].

Effective telemedicine services depend on the acceptance and satisfaction of both healthcare users and service providers [8-10]. During the COVID-19 pandemic, a simplified telemedical setup was easy and was widely accepted by diabetic patients and healthcare providers [11]. In ophthalmology, remote consultation is challenging due to the lack of biomicroscopic assessment, as well as the complex investigations needed for diagnosis and management. However, teleophthalmology could be convenient when reviewing previously diagnosed patients.

Several reports have studied patients’ satisfaction with virtual eye care clinics [8,12,13]. However, little is known about ophthalmologists’ satisfaction with virtual ophthalmology clinics and whether the subspecialty of ophthalmologists plays a role.

The purpose of this study is to evaluate the level of satisfaction and its determinants among ophthalmologists who participated in Virtual Ophthalmology Clinics (VOCs) during the COVID-19 pandemic at a tertiary eye care institute in central Saudi Arabia.

## Materials and methods

Study design and population

A physician-based, descriptive, cross-sectional study was conducted following the tenets of the Declaration of Helsinki at a referral tertiary care eye hospital in central Saudi Arabia. The study was conducted between November 2020 and September 2021. Ethical approval for the study was obtained from the Institutional Review Board of King Khaled Eye Specialist Hospital (number: 20140-P). All participating ophthalmologists provided written informed consent before the commencement of the study.

The inclusion criteria were all ophthalmologists who participated in VOCs during the COVID-19 pandemic in King Khaled Eye Specialist Hospital (n = 113), whereas those with no experience with VOCs were excluded. The sampling technique used in this study was a convenient sampling technique in which all eligible participants were included.

Assessment tool and statistical analysis

A questionnaire was formulated for assessing ophthalmologists’ satisfaction with VOCs and was sent to their e-mail addresses. The questionnaire included domains related to their satisfaction with the scheduling process, equipment and technical issues, communication, clinical assessment, and the overall program evaluation.

The questionnaire comprised 18 questions with five-point Likert scale categories ranging from “strongly disagree” coded as 1 to “strongly agree” coded as 5, with a Cronbach alpha of 0.815, or 81.5%, indicating very good internal consistency. Thus, the questionnaire was valid for use in this study.

After the data were extracted, SPSS version 26 (IBM Corp., Armonk, NY, USA) was used for data analysis. The overall satisfaction score was obtained by adding all 18 questions, and a score ranging from five to 90 points was generated, indicating that the higher the score, the higher the satisfaction with virtual consultations. By using 60% as a cut-off point to determine the level of satisfaction, ophthalmologists were classified as dissatisfied if the score was 60% or below, and those above 60% were classified as satisfied. Negative questions were re-coded reversely to avoid bias in the overall score.

Descriptive statistics were presented using numbers, percentages, mean, and standard deviation whenever appropriate. The differences in satisfaction scores according to subspecialty, the effect of staff training, and prior experience with VOCs were investigated using an independent sample t-test and one-way analysis of variance (ANOVA) with corresponding values for statistical tests such as t-test (independent samples) and F-test (one-way ANOVA). The normality test was performed using the Shapiro-Wilk test as well as the Kolmogorov-Smirnov test. The satisfaction score follows a normal distribution (p > 0.05). Thus, a parametric test was applied when conducting statistical tests. Post hoc analysis was also performed to determine the multiple mean differences using Tukey’s honestly significant difference (Tukey’s HSD) test. P-values of <0.05 were considered as the cut‑off value for significance.

## Results

Of the total 113 ophthalmologists who experienced VOCs, 71 (62.8%) completed the study questionnaire. The basic demographic characteristics of the participating ophthalmologists are summarized in Table 1. The most common age group was 21-30 years old (42.3%), with two-thirds of participants being males (66.2%). Most of the surveyed ophthalmologists were general ophthalmologists (32.4%), followed by glaucoma specialists (22.5%), cornea specialists (21.1%), retina specialists (11.5%), and pediatric ophthalmologists (9.9%). In total 30 (42.3%) of the respondent ophthalmologists had a clinical experience of fewer than five years, while 25 (35.2%) had a clinical experience of 5-10 years and 14 (19.7%) had an experience of 10 years or more.

**Table 1:** Demographic characteristics of the ophthalmologists who experienced VOCs during the COVID-19 pandemic. VOCs: Virtual Ophthalmology Clinics; COVID-19: coronavirus disease 2019

Study data	N (%)
Age group (years)	
21–30	30 (42.3%)
31–40	24 (33.8%)
41–50	8 (11.3%)
51–60	7 (9.9%)
>60	2 (2.8%)
Gender	
Male	47 (66.2%)
Female	24 (33.8%)
Years in practice (years)	
<5	30 (42.3%)
5–10	25 (35.2%)
11–15	2 (2.8%)
>15	14 (19.7%)

The domains of the Virtual Ophthalmology Follow-up Visits questionnaire are reported in Table 2 which comprised four domains, including equipment/technical issues, communication and rapport, clinical assessment, and overall perception of virtual consultations. For equipment/technical issues (three items), the overall mean score was 9.80 (SD = 2.23). The mean score was higher in the statement “I’m satisfied with the quality of audio consultations” (mean = 3.39), while the mean score was lower in the statement “I’m satisfied with the quality of video consultations” (mean = 3.03). For the communication and rapport domain (four items), the overall mean score was 13.3 (SD = 3.26). The mean score was higher in the negative question “I feel that virtual consultations negatively impact patient privacy” (mean = 3.87), while it was lower in the statement “I am satisfied with the level of clinician-patient rapport that I can achieve with virtual consultations” (mean = 3.03). For the clinical assessment domain (six items), the overall mean score was 16.9 (SD = 5.15). The mean score was higher in the statement “Virtual consultations provide desirable results in patient management” (mean = 3.15), while it was lower in the negative question “I can not competently assess, treat patients through virtual consultations to the extent that I believe is required” (mean = 2.48). Finally, for the overall opinion about virtual consultations (five items), the overall mean score was 16.3 (SD = 4.24). The mean score was higher in the statement “Virtual consultations were highly utilized during the COVID-19 pandemic” (mean = 4.03), while it was lower in the statement “I would prefer using virtual consultations for future follow-ups with my patients” (mean = 2.68). Based on the 18-item satisfaction questionnaire, the overall mean satisfaction score was 56.3 (SD = 12.7), with 43.7% classified as dissatisfaction level and 56.3% classified as satisfaction level.

**Table 2:** Domains of Virtual Ophthalmology Follow-up Visits questionnaire. ^†^Indicate negative questions. Responses range from “strongly disagree” coded as 1 to “strongly agree” coded as 5. SD: standard deviation; COVID-19: coronavirus disease 2019

Statement	Mean ± SD
Equipment/Technical issues	9.80 ± 2.23
1. Find the virtual consultation system easy to use	3.38 ± 0.92
2. I am satisfied with the quality of audio consultations	3.39 ± 0.96
3. I am satisfied with the quality of video consultations	3.03 ± 0.89
Communication and rapport	13.3 ± 3.26
4. I am satisfied with the level of clinician-patient rapport that I can achieve with virtual consultations	3.03 ± 1.13
5. I feel that virtual consultations negatively impact patient privacy^†^	3.87 ± 0.99
6. I feel that patients can easily follow my instructions during virtual consultation appointments	3.17 ± 0.97
7. Interaction and communication with patients are good enough to convey medical messages	3.25 ± 1.02
Clinical assessment	16.9 ± 5.15
8. I can assess the current status of my patients’ condition	2.77 ± 1.21
9. I cannot competently assess and treat patients through virtual consultations to the extent that I believe is required^†^	2.48 ± 1.22
10. Virtual consultations provide desirable results in patient management	3.15 ± 0.95
11. I do not feel comfortable implementing virtual clinic consultations^†^	2.85 ± 1.24
12. I believe that I can decide the appropriate next step in the management of my patients (surgery/procedure/medications)	2.65 ± 1.19
13. I am able to predict the prognosis of my patients through virtual consultations	2.99 ± 1.14
Overall opinion about virtual consultations	16.3 ± 4.24
14. Virtual consultations were highly utilized during the COVID-19 pandemic	4.03 ± 1.03
15. I was confident in implementing patients’ consultations virtually	3.28 ± 1.08
16. Overall, I am satisfied with the clinical outcome I can achieve via virtual consultations	3.04 ± 1.11
17. Overall, I am satisfied with the level of virtual service provided to my patients	3.24 ± 1.02
18. I prefer using virtual consultations for future follow-ups with my patients	2.68 ± 1.14
Overall score	56.3 ± 12.7
Level of satisfaction	
Dissatisfied	31 (43.7%)
Satisfied	40 (56.3%)

The effect of subspecialty, years in practice, and prior experience with VOC and its correlation to their overall satisfaction level is demonstrated in Table 3. The satisfaction score of the retina subspecialty was statistically significantly higher than the other subspecialties (F = 9.716; p < 0.001). Furthermore, only nine (12.7%) ophthalmologists had a history of training in teleophthalmology. In total, 12 (16.9%) study ophthalmologists reported providing teleophthalmology before the pandemic, while 52 (73.2%) did not manage any teleophthalmology at that time. During COVID-19, 15.5% of the ophthalmologists reported that they conducted video consultations with patients, 53 (74.6%) reported conducting phone consultations with patients, and 23 (32.4%) conducted consults with other healthcare providers that included photographs or videos provided in person, through e-mail, or online more than five times. Ophthalmologists who conducted a video consultation more than five times during the pandemic showed significantly better satisfaction scores than the others (F = 5.766; p = 0.001). On the other hand, the differences in satisfaction scores of years in practice, received training in teleophthalmology, provided teleophthalmology before the pandemic, provided phone consultations during the pandemic, and conducted consultations with other healthcare providers were not significantly different (p > 0.05).

**Table 3:** The effect of subspecialty, years in practice, and prior experience with VOCs and its correlation to their overall satisfaction level. ^a^P-value has been calculated using one-way ANOVA test. ^b^P-value has been calculated using independent sample t-test. ^**^Significant at p<0.05 level. VOCs: Virtual Ophthalmology Clinics; COVID-19: coronavirus disease 2019

Factor	Satisfaction score (90), mean ± SD	T/F-test	P-value
Subspecialty^a^			
General ophthalmology	56.2 ± 10.4	F = 9.716	<0.001**
Cornea	51.3 ± 7.79		
Glaucoma	49.7 ± 12.1		
Pediatrics	56.4 ± 12.5		
Retina	75.9 ± 7.99		
Years in practice^a^			
<5	54.3 ± 10.6	F = 3.141	0.069
5–10	54.3 ± 10.2		
>10	63.1 ± 17.4		
Received training in teleophthalmology^b^			
Yes	56.5 ± 12.8	T = 0.407	0.685
No	54.7 ± 12.3		
Conducted teleophthalmology before the pandemic^b^			
Yes	59.4 ± 10.9	T = 1.246	0.217
No	55.2 ± 13.2		
Conducted video consultations during the COVID-19 pandemic^a^			
None	53.0 ± 10.6	F = 5.766	0.001 **
Less than three times	59.4 ± 6.74		
Three to five times	52.6 ± 24.6		
More than five times	68.5 ± 10.7		
Conducted phone consultations during the COVID-19 pandemic^a^			
None	57.7 ± 13.9	F = 0.144	0.933
Less than three times	58.8 ± 4.55		
Three to five times	57.4 ± 11.4		
More than five times	55.7 ± 13.3		
Conducted consults with other healthcare providers that included photographs or videos provided in person, through e-mail, or online^a^			
None	52.9 ± 11.6	F = 1.577	0.203
Less than three times	60.6 ± 10.1		
Three to five times	55.4 ± 11.9		
More than five times	59.5 ± 14.4		

We then performed a post-hoc analysis to determine the multiple mean differences in the satisfaction score according to subspecialty (Table 4). Based on the results, there was a significant difference in the mean score between general ophthalmology and retina subspecialty (p = 0.001), cornea and retina (p = 0.001), and glaucoma and retina (p = 0.001). Further, there was a significant difference in the satisfaction score between pediatric ophthalmology and retina subspecialty (p = 0.005).

**Table 4:** Post-hoc analysis to determine the multiple mean differences in ophthalmologists’ satisfaction based on their subspecialties (n = 71). ^§^P-value has been calculated using Tukey’s HSD. **Significant at p<0.05 level. HSD: honestly significant difference

Subspeciality (I)	Subspeciality (J)	Mean difference (I-J)	Standard error	95% CI	P-value^§^
General ophthalmology	Cornea	4.8	3.4	-4.8 to 14.4	0.620
	Glaucoma	6.5	3.4	-2.9 to 15.9	0.310
	Pediatric ophthalmology	-0.3	4.4	-12.7 to 12.2	1.000
	Retina	-19.7	4.2	-31.6 to -7.8	<0.001**
Cornea	General ophthalmology	4.8	3.4	-14.4 to 4.8	0.620
	Glaucoma	1.6	3.7	-8.7 to 12.0	0.992
	Pediatric ophthalmology	-5.1	4.7	-18.3 to 8.1	0.816
	Retina	-24.5	4.5	-37.2 to -11.9	<0.001**
Glaucoma	General ophthalmology	-6.5	3.4	-15.9 to 2.9	0.310
	Cornea	-1.6	3.7	-12.0 to 8.7	0.992
	Pediatric ophthalmology	-6.7	4.7	-19.8 to 6.4	0.602
	Retina	-26.2	4.5	-38.7 to -13.7	<0.001**
Pediatric ophthalmology	General ophthalmology	0.3	4.4	-12.2 to 12.7	1.000
	Cornea	5.1	4.7	-8.1 to 18.3	0.816
	Glaucoma	6.7	4.7	-6.4 to 19.8	0.602
	Retina	-19.4	5.3	-34.4 to -4.5	0.005**
Retina	General ophthalmology	19.7	4.2	7.8 to 31.6	<0.001**
	Cornea	24.5	4.5	11.9 to 37.2	<0.001**
	Glaucoma	26.2	4.5	13.7 to 38.7	<0.001**
	Pediatric ophthalmology	19.14	5.3	4.5 to 34.4	0.005**

**Figure 1:**
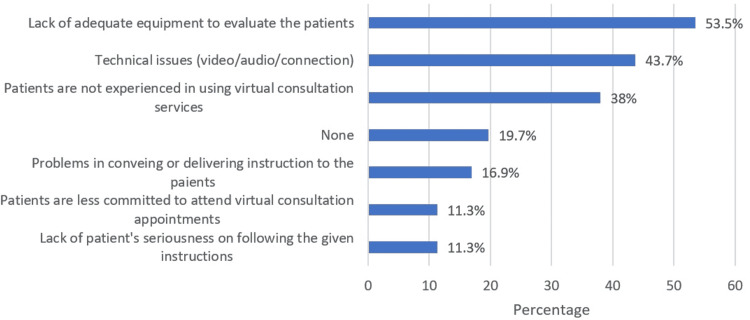
Challenges encountered during the Virtual Ophthalmology Clinic.

The most common barriers encountered during virtual consultation were the lack of adequate equipment to evaluate the patients (53.5%), followed by technical issues (43.7%) and the patients’ lack of experience in using virtual consultation services (38%) (Figure 1).

## Discussion

The present study was designed to evaluate the level of ophthalmologists’ satisfaction with their VOC experience in a tertiary eye care institute in central Saudi Arabia. Since 2019, COVID-19 has spread rapidly, resulting in considering the disease as a pandemic in 2020. The World Health Organization (WHO) guidelines endorse social distancing and quarantine to prevent disease transmission [14,15]. Pandemic restrictions have led to an increase in the utilization of virtual patient visits between 257% and 700%, after approximately 74% of patients were unaware of the concept of teleconsultation [16]. In Saudi Arabia, Albarrak et al. reported that the implementation of telemedicine technologies helps improve access to healthcare and improves its quality for both patients and ophthalmologists [17]. Therefore, measuring the level of ophthalmologists’ satisfaction is an important issue when considering future implementation of VOCs in Saudi Arabia. This study is one of the first quantitative studies to evaluate the level of ophthalmologists’ satisfaction in the Middle East.

Our results revealed that 31 ophthalmologists were poorly satisfied, while 40 ophthalmologists had a high level of satisfaction regarding the use of VOCs. These findings are in line with an American study by Kirby et al. where most ophthalmologists were either “satisfied” (4/5) or “very satisfied” (5/5) (75.0%) [18]. Despite the advantages of remote consultation, there is no standardization yet for patients’ eye assessment during virtual care. In our questionnaire, 45.1% of the ophthalmologists felt they could not adequately assess the current status of their patients’ conditions, and 38.0% felt they could not predict the prognosis of their patients’ conditions. These findings broadly support other studies in this field, making the inability to perform adequate ophthalmic examinations a major barrier, if not the only one, to the long-term utilization of [19,20]. Although expected, a reliable method of examining patients’ eyes is needed to maintain this as a viable method of care.

Training healthcare workers is crucial to the effectiveness of telemedicine, most effectively achieved by recorded videos and live webinars. Healthcare workers can watch the recorded video training as much as they want and have their commonly asked questions answered [21]. In this study, we investigated the effect of ophthalmologists’ training and its correlation with their satisfaction. Our data indicated that 87.3% of the ophthalmologists in our study did not receive training in telemedicine, whereas 12.7% of the ophthalmologists did. Of those who received training on telemedicine, a mean satisfaction score of 56.5 was reported. However, the differences in the satisfaction scores of receiving training in telemedicine were not significantly different (p > 0.05). These findings have implications for the importance of staff training before using VOCs.

Years of clinical practice play a role in ophthalmologists’ satisfaction with VOCs. An orthopedic study conducted by Neville et al. reported that years in clinical practice were not statistically associated with the likelihood of offering telemedicine visits post-COVID-19 [22]. Similarly, 42.3% of ophthalmologists had less than five years of practice experience in our study. In Table 3, the differences in the satisfaction score of years in practice were not significantly different (p > 0.05). More research is needed to develop a complete picture of the relationship between years of practice and the level of satisfaction with VOCs.

The present study is significant in at least two major respects. Both the subspecialty of the ophthalmologist and how much the ophthalmologist conduct video consultations might affect the overall experience of VOCs. Due to limited literature, there is very little information available about the experience and perception of ophthalmologists with telemedicine. In this study, we explored the influence of subspecialty and its relationship with the satisfaction score. Among ophthalmology subspecialties, retina subspeciality reported the highest satisfaction scores (75.9). Pediatric ophthalmology reported the second-highest satisfaction score (56.4). A recent article investigating the experience of pediatric ophthalmologists reported a low confidence level in telemedicine to deliver eye care [20]. This difference in perceptions among pediatric ophthalmologists could be due to different pandemic peaks at the time of research.

The strength of our study included a response rate of 62.8%, which is higher than expected for e-mail-based physician surveys (which typically have a response rate of less than 30%) [23,24]. In addition, our study included ophthalmologists from different subspecialties (general, glaucoma, cornea, retina, and pediatric ophthalmology).

Limitations of this study include the small sample size and the short period of virtual clinic experience as it is possible for ophthalmologists’ perceptions and satisfaction levels to change as COVID-19 severity fluctuates and restrictions ease. Additionally, our study design did not allow drawing causal inferences, and only descriptive statistics were presented. Hence, the associations could be biased due to confounding.

## Conclusions

The emergence of the COVID-19 pandemic posed an unexpected challenge in delivering healthcare through conventional clinics due to social distancing regulations. One of the temporary solutions was the utilization of virtual clinics. We believe that the pandemic significantly impacted eye care delivery as ophthalmic examinations are challenging to perform virtually. In our survey, more than half of the respondents were satisfied with teleophthalmology, with retina specialists reporting the highest level of satisfaction. The frequency of video consultations was found to be an important factor in ophthalmologists’ satisfaction with teleophthalmology. Fortunately, the current pandemic could be an opportunity to further improve ophthalmology telemedicine services, especially for those who live far away from the hospital and therefore will not require further examinations. In addition, greater effort is needed to develop and standardize a method for patients’ eye examinations.
